# Single-cell RNA sequencing of equine mesenchymal stromal cells from primary donor-matched tissue sources reveals functional heterogeneity in immune modulation and cell motility

**DOI:** 10.1186/s13287-020-02043-5

**Published:** 2020-12-04

**Authors:** Rebecca M. Harman, Roosheel S. Patel, Jennifer C. Fan, Jee E. Park, Brad R. Rosenberg, Gerlinde R. Van de Walle

**Affiliations:** 1 Baker Institute for Animal Health, College of Veterinary Medicine, Cornell University, Ithaca, NY 14853 USA; 2 Department of Microbiology, Icahn School of Medicine at Mount Sinai, New York, NY 10029 USA

**Keywords:** Mesenchymal stromal cells, Single-cell RNA sequencing, Heterogeneity, Immune modulation, Cell motility

## Abstract

**Background:**

The efficacy of mesenchymal stromal cell (MSC) therapy is thought to depend on the intrinsic heterogeneity of MSC cultures isolated from different tissue sources as well as individual MSCs isolated from the same tissue source, neither of which is well understood. To study this, we used MSC cultures isolated from horses. The horse is recognized as a physiologically relevant large animal model appropriate for translational MSC studies. Moreover, due to its large size the horse allows for the simultaneous collection of adequate samples from multiple tissues of the same animal, and thus, for the unique collection of donor matched MSC cultures from different sources. The latter is much more challenging in mice and humans due to body size and ethical constraints, respectively.

**Methods:**

In the present study, we performed single-cell RNA sequencing (scRNA-seq) on primary equine MSCs that were collected from three donor-matched tissue sources; adipose tissue (AT), bone marrow (BM), and peripheral blood (PB). Based on transcriptional differences detected with scRNA-seq, we performed functional experiments to examine motility and immune regulatory function in distinct MSC populations.

**Results:**

We observed both inter- and intra-source heterogeneity across the three sources of equine MSCs. Functional experiments demonstrated that transcriptional differences correspond with phenotypic variance in cellular motility and immune regulatory function. Specifically, we found that (i) differential expression of junctional adhesion molecule 2 (*JAM2*) between MSC cultures from the three donor-matched tissue sources translated into altered cell motility of BM-derived MSCs when RNA interference was used to knock down this gene, and (ii) differences in C-X-C motif chemokine ligand 6 (*CXCL6*) expression in clonal MSC lines derived from the same tissue source correlated with the chemoattractive capacity of PB-derived MSCs.

**Conclusions:**

Ultimately, these findings will enhance our understanding of MSC heterogeneity and will lead to improvements in the therapeutic potential of MSCs, accelerating the transition from bench to bedside.

**Supplementary Information:**

The online version contains supplementary material available at 10.1186/s13287-020-02043-5.

## Background

The potential of mesenchymal stromal cells (MSCs) for therapeutic use has been an active area of research for several decades [1–4]. Thousands of studies, both in vitro and in animal models, have been published, providing evidence for the therapeutic value of MSC, particularly as immunomodulatory cells that migrate to injured tissues and contribute to repair [5–7]. This work has led to the initiation of hundreds of human clinical trials, but despite promising results, MSCs are not widely used therapeutically [8, 9]. This slow transition of MSC therapy from bench to bedside can, in part, be explained by inconsistent study results due to variation in MSC cultures [10].

MSCs can be isolated from various tissue sources including, but not limited to, adipose tissue (AT), bone marrow (BM), peripheral blood (PB), placenta, and dental pulp [11, 12]. The resulting cultures derived from different tissue sources typically exhibit varying degrees of cellular heterogeneity (inter-source heterogeneity). In addition, mixed populations of cell types are suspected to make up cultures isolated from single tissue sources (intra-source heterogeneity). MSC heterogeneity is traditionally assessed by morphology, expression of surface markers, cell kinetics, differentiation potential, and select gene expression patterns [13, 14]. However, the transcriptional heterogeneity of MSCs remains largely unexplored and its contribution to the therapeutic value of MSCs is poorly understood [15].

Single-cell RNA sequencing (scRNA-seq) is a technology that profiles single cells based on gene expression patterns and has been extensively used to characterize cellular diversity in human and mouse models [16, 17]. Studies presenting scRNA-seq datasets to address inter-source variation of MSCs have identified unique gene expression patterns when primary human Wharton’s Jelly-derived MSC were compared with commercial human BM-derived MSCs [18], and when primary human umbilical cord-derived MSCs from one donor set were compared with human synovial fluid-derived MSC collected from a separate set of patients [19]. Ideally, inter-source heterogeneity should be explored by evaluating samples isolated from the same individual to exclude donor-to-donor variation. However, it is difficult to collect multiple tissue specimens from the same human donor due to ethical constraints, especially if tissue collection is not required for routine care and/or if the collection procedure is invasive, as is the case for BM and AT samples. One study, using scRNA-seq and functional follow-up experiments, explored donor-matched human MSCs derived from two sources (BM, AT) and found that AT-derived MSCs depend less on mitochondrial respiration for energy production, express lower levels of human leukocyte antigen class I (MHC Class I), and have an increased immunosuppressive capacity relative to BM-derived MSC [20]. To date, there are no reports on scRNA-seq data on donor-matched tissue-derived murine MSCs. Current scRNA-seq datasets generated to address the intra-source variation of MSC have led to various outcomes. One study reported a lack of heterogeneity in human AT-derived MSCs [21], whereas a later study found them to be heterogeneous [22]. Other studies reported heterogeneity within murine BM-derived MSC and based on gene expression patterns, proposed this to be important for creating specific niches for maintenance and lineage priming of immune cells [23, 24].

To overcome the practical hurdles of collecting donor matched MSCs from different tissue sources, the horse can be used as a model as its large size allows for the collection of adequate samples from multiple tissues without compromising the health of the animal. Additional advantages of using the horse as a model are (i) the availability of an annotated horse genome, (ii) unlike many laboratory animals, the horse being an outbred species like humans, and (iii) the horse being a relevant translational model for the evaluation of novel stem cell-based therapies [25–27].

In this study, we applied scRNA-seq to explore the inter- and intra-source heterogeneity of primary equine MSCs collected from three donor-matched tissue sources (AT, BM, PB). Functional experiments demonstrated that detected transcriptional differences correspond with phenotypic variation in cellular motility and immune regulatory function. This improved understanding of MSC heterogeneity will lead to improvements in the potential of MSC therapy, accelerating its transition from bench to bedside.

## Methods

### Cells

Equine mesenchymal stromal cells (MSCs) used for single-cell RNA sequencing (scRNA-seq) were isolated and cultured from adipose tissue (AT), bone marrow (BM) and peripheral blood (PB), of a healthy adult research warmblood mare, euthanized for reasons unrelated to this study, exactly as described previously [28]. MSC used for validation of gene expression patterns were isolated and cultured from AT, BM and PB of two healthy adult research thoroughbred geldings, euthanized for reasons unrelated to this study. Equine MSCs were characterized by immunophenotypical protein profiling using flow cytometry and the potential for trilineage differentiation, exactly as described previously [28, 29]. Equine neutrophils were isolated from equine peripheral blood, exactly as previously described [30], and used in chemotaxis assays immediately following isolation. Blood collection was approved by the Cornell Institutional Animal Care and Use Committee (IACUC # 2014–0038). Equine vascular endothelial cells (EC) were isolated from the *a. carotis* of healthy horses, euthanized for reasons not related to this study, and cultured, exactly as described before [31].

### Single cell library preparation and sequencing

After two passages in culture, MSCs were processed for scRNA-seq on the 10X Genomics Chromium platform (10X Genomics). Each sample was processed in a single lane on the 10X Genomics Chromium instrument, with a targeted cell recovery of 5000 cells per sample. scRNA-Seq libraries were prepared with the 10X Genomics Chromium Single Cell 3′ Reagent Kit (v2), according to manufacturer’s instructions. Libraries were pooled and sequenced on the Illumina NextSeq 500 in paired-end configuration (Read 1, cell barcode: 26 nt; Read 2, transcript: 98 nt) to a read depth of approximately 24,000 paired-end reads per cell.

### scRNA-seq data processing

The EquCab3.0 reference genome [32] was used in all analyses. Reads were assigned to cell barcodes, mapped and quantified per gene using CellRanger (v 3.0.1, 10X Genomics) with default parameters (“standard workflow”). Raw BAM files were extracted and processed with the End Sequence Analysis Toolkit [33] and a workflow optimized for equine single cell RNA-seq analysis as described in Patel et al. 2020 [34]. Briefly, extracted BAM files were modified such that cell barcode and UMI information were appended to the corresponding read name entry for processing by ESAT. ESAT evaluates reads mapped immediately downstream of annotated genes and includes these reads in expression quantification of the associated gene. Overlapping gene intervals (exons from two separate genes on + and – strands sharing the same and/or overlapping genomic coordinates) were excluded from expression quantification. To recover expression of features duplicated (genetic elements that had identical sequences represented in 2 or more regions in the in the EquCab3.0 genome reference), ESAT was run a second time, with the parameter “–multimap scale”, on a filtered reference containing only duplicated features. The resulting (duplicated) gene x cell count matrices were “flattened” to single entries per gene and appended to the primary gene x cell count matrix. Putative “multiplet” cell barcodes were identified and removed from downstream analyses with the DoubletDetection tool [35].

### scRNA-seq data analysis

Processed gene-cell matrices were analyzed in the R statistical environment (v3.5.1) using the Seurat package (v3.1.1). Data were filtered to exclude genes detected in less than 3 cells (per tissue source), to exclude cells with less than 2500 unique molecular identifiers (UMIs) or greater than 15,000 UMIs (putative doublets), and to exclude cells with greater than 3% UMIs assigned to mitochondrial genes (putative dead or dying cells). Gene-cell count matrices were independently normalized with SCTransform [36], and the top 3000 most variable genes (variance-stabilizing transformation) were selected for dimensional reduction by principal component analysis (PCA). Seurat’s CellCycleScoring function was used to score and identify cell cycle phase (G1, G2M or S) of individual cells. During normalization, cell cycle score differences (G2M-S) and mitochondrial transcript percentages were included as factors for regression. Unsupervised graph-based clustering (smart local moving algorithm [37], resolution 0.6) was performed on the first 14 (AT-MSC), 23 (BM-MSC), 20 (PB-MSC) principal components (selected by Scree plot visualization). Data annotated with corresponding clusters were visualized by Uniform manifold approximation and projection (UMAP; n.dims: as for clustering, n.neighbors: 30, cosine metric, min.dist: 0.3) [38]. Differential gene expression analyses were conducted using edgeR (v3.26.8) [39], with additional modifications for scRNA-seq data [40]. Gene expression linear models included factors for cellular gene detection rate (to account for scRNA-seq “dropout”), cluster, and cell cycle score differences (as identified in Seurat analysis above). Specific contrasts are detailed in relevant Results sections and/or figures. For all differential gene expression testing analyses, genes expressed (i.e. greater than or equal to 1 UMI) in less than 25% of cells for at least one cluster/group within a contrast were excluded from differential expression results. DGE tables were further filtered to only include genes with an adjusted *p* value < 0.05. Gene Ontology enrichment analysis was conducted with the goana function, and biological process GO terms with a p value < 0.0005 were reported in results.

### siRNA knockdown

Silencer Select siRNAs targeting equine junctional adhesion molecule 2 (*JAM2)* were designed (Thermo Fisher, 4,399,665). Silencer Select Negative Control #2 siRNA was confirmed to not have complementarity to any equine genes using BLAST (https://blast.ncbi.nlm.nih.gov/Blast.cgi), and was used as a non-specific (scramble) control. For RNA-mediated interference (RNAi), 1.25 × 10^4^ MSCs were seeded per cm^2^. Lipofectamine RNAiMAX Reagent (Thermo Fisher) siRNA complexes were generated, incubated for 5 min, and used to transfect cells with 5 nM siRNA. Lipofectamine:siRNA ratios varied based on culture well size, according to manufacturer’s recommendations.

### Reverse transcription–polymerase chain reaction (RT-PCR)

SYBR green–based RT-PCR was performed to determine fold change in transcripts of interest, and data were analyzed, as previously described [41, 42]. The previously validated reference gene beta-2-microglobulin *(BM2)* was used to normalize samples [43] and all samples, were run in triplicate. Genes and primer sequences are listed in Table 1.

**Table 1:** Primers used for RT-PCR

Gene Product	Abbreviation	Forward primer (5′-3′)	Reverse primer (5′-3′)
junctional adhesion molecule 2	*JAM2*	AAAGTTGGCTCCCAAAGCAC	ACACTTGCGATGTCCAACAG
C-X-C motif chemokine ligand 6	*CXCL6*	AGAGAACT GCGTTGCATGTG	TCAAGGTGGCTACGACTTCC
adrenomedullin	*ADM*	TCCCGTAACCCTCATGTACC	AAGTTCCCTCTTCCCACGAC
insulin-like growth factor binding protein 5	*IGFBP5*	CTCATTATTCCGGTGGTTGC	GTGGAGGCTGGAGAGACAAG
monoglyceride lipase	*MGLL*	AAAGGAGCCTACTTGCTCATGG	TTTCACGGAAGACGGAGTTG
asporin	*ASPN*	AAACCCTTGCTTCACCCTTC	TCAGCGTCACTGTCACCTTC
cellular retinoic acid binding protein 2	*CRABP2*	CCTGGTGAAATGGGAAAGTG	TGTCGTCCGCTGTCATAGTC
beta-2-microglobulin	*B2M*	TCTTTCAGCAAGGACTGGTCTTT	CATCCACACCATTGGGAGTAAA

### Western blotting

Western blotting was used to determine the efficacy of JAM2 silencing in BM-MSC by siRNA, as previously described [42]. Primary antibodies were rabbit anti-JAM2 (ab96465) and rabbit anti-β-actin (ab8227) (Abcam, Cambridge, MA) as loading control, each diluted 1:1000. Images of blots were captured on a Bio-Rad imaging system (Bio-Rad, Hercules, CA) and JAM2 band intensity relative to β-actin band intensity was determined using ImageJ image processing and analysis software (https://imagej.nih.gov/ij/).

### Proliferation, adhesion, invasion, and migration assays

*To asses proliferation, a* 5-Bromo-2′-deoxyuridine (BrdU) incorporation ELISA (Abcam) was used, as previously described [43]. Cell adhesion was determined using a microcentrifugation assay, as previously described [42]*. *MSC invasion through a monolayer of EC was determined by electric cell-substrate impedance sensing (ECIS), as previously described [44]*. To asses migration,* in vitro scratch assays with MSCs were performed in 12-well plate wells, as previously described [45]. Photographs of scratches were taken at 0 and 48 h post-scratching, and migration distances of cells were calculated in a blinded manner using ImageJ software (http://imagej.nih.gov/ij/).

### Cloning

Cloning of MSCs was carried out, as described before [46], by plating an average of 0.5 cells labeled with CellTracker Green (Thermo Fisher, Waltham, MA) per well in 96-well cell culture plates in expansion medium. Four 96-well plates (192 cells) were initially plated. Wells were observed and those containing one cell were identified and labeled. After 7–10 days, single cells formed colonies that were considered clones. Fourteen clones were first expanded to 12-well plate wells and then T75 tissue culture flasks, before being frozen for RNA extraction and further culture experiments (Figure S1).

### Chemotaxis assays

PB-derived MSC clones were plated in triplicate wells of 24-well plates fitted with coverslips, at a density of 100,000 cells per well in expansion medium. After 24 h, medium was removed, cell monolayers were rinsed with PBS, and 2 ml DMEM was added to each well. The next day, conditioned medium (CM), containing all factors secreted by MSC clones, was collected by removing DMEM from culture wells and centrifuging twice at 300 x g for 10 min at room temperature (RT) to remove cellular debris. Six hundred μl CM was transferred to a well of 24-well plates containing coverslips, in triplicate. Each well was then fitted with an insert containing a 3 μm filter (Corning, Corning, NY). Equine neutrophils were plated in each insert at a density of 20,000 cells per insert. Cultures were incubated for 1 h at 37 °C at which time inserts were removed, and plates were centrifuged at 200 x g for 5 min at RT. Culture medium was gently removed, coverslips were rinsed with PBS, and adherent cells were fixed with 70% ethanol before staining with hematoxylin (Sigma Aldrich). Coverslips were mounted on slides and photographed. Neutrophils in 10 random fields were counted and quantified.

### Statistical analysis

Chemotaxis, proliferation, adhesion, invasion, migration, and RT-PCR assays were run in triplicate and analyzed by an ordinary one-way ANOVA, followed by a Tukey’s multiple comparisons test. GraphPad Prism (GraphPad Software, San Diego, CA, www.graphpad.com) was used for analysis, *P* < 0.05 was considered significant.

## Results

### Single-cell RNA sequencing (scRNA-seq) data reveal inter-source variation of equine mesenchymal stromal cells (MSCs) isolated from donor-matched tissue sources

**Fig. 1:**
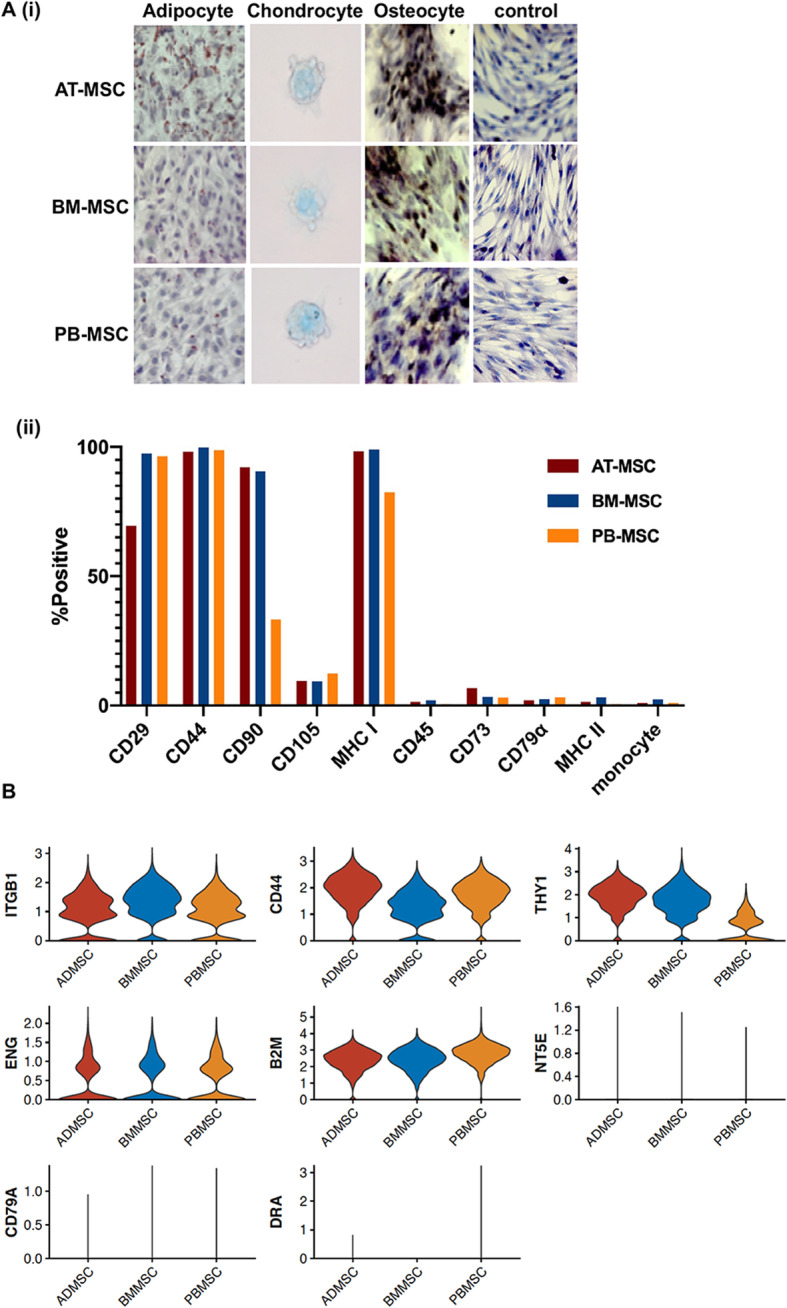
Characterization of equine mesenchymal stromal cells (MSCs) isolated from 3 donor-matched tissue sources. **a** 40x images of adipose tissue (AT-), bone marrow (BM-) and peripheral blood (PB-) derived MSC after in vitro differentiation into adipocytes (oil red O), chondrocytes (alcian blue) and osteocytes (alizarin red). Undifferentiated cells are shown as controls (hematoxylin) **(i)** and cellular expression patterns of proteins determined by the International Society for Cellular Therapy (ISCT) to be used for MSC immunophenotyping, as detected by flow cytometry **(ii)**. **b** sc-RNAseq violin plots of the expression levels of the genes corresponding to proteins used for MSC characterization

Equine MSCs were isolated from donor-matched adipose tissue (AT), bone marrow (BM), and peripheral blood (PB), and characterized based on their potential to differentiate into adipocytes, chondrocytes and osteocytes as well as expression patterns of cellular proteins, as recommended by the International Society for Cellular Therapy (ISCT) [47]. This characterization corroborated what we and others previously found for equine MSCs [28, 45, 48], and as historically reported, little to no inter-, or intra-, source variation in MSCs was observed [48] (Fig. 1a). scRNA-seq data further supported the classification of the equine cells isolated from the 3 different sources as MSCs. For example, MSC marker genes *ITGB1* (CD29), *CD44, THY* (CD90), *ENG* (CD105) and *B2M* (a subunit of MHC Class I) were expressed by the majority of cells from each source (Fig. 1b), corroborating our flow cytometry findings (Fig. 1a(ii)). Moreover, transcripts for *NT5E* (CD73), *CD79A,* and *DRA* (MHC Class II) genes were hardly detected in the analyzed cells (Fig. 1b) and transcripts for the gene *PTPRC* (CD45) were not represented at all. Although we cannot rule out that absence of transcript counts could be due to incomplete sampling depth inherent to droplet scRNA-Seq technology, these data align with the corresponding protein expression patterns we observed (Fig. 1a(ii), S2). Although CD73 is proposed by the ISCT as a positive marker of human MSC, we and others previously reported on its inconsistent and even absent expression in equine MSC [25, 45, 48]. According to ISCT definitions, MSCs do not express the endothelial cell marker CD34 [47]. In agreement with this, we did not detect *CD34* transcripts in appreciable numbers of equine MSC isolated from any of the three MSC sources, with the exception of a few transcripts in a minority of cells in AT-derived MSC (Figure S3A). Unfortunately, CD34 protein expression cannot be evaluated in equine MSC due to the lack of cross-reacting and/or commercially available equine-specific anti-CD34 antibodies [48].

**Fig. 2:**
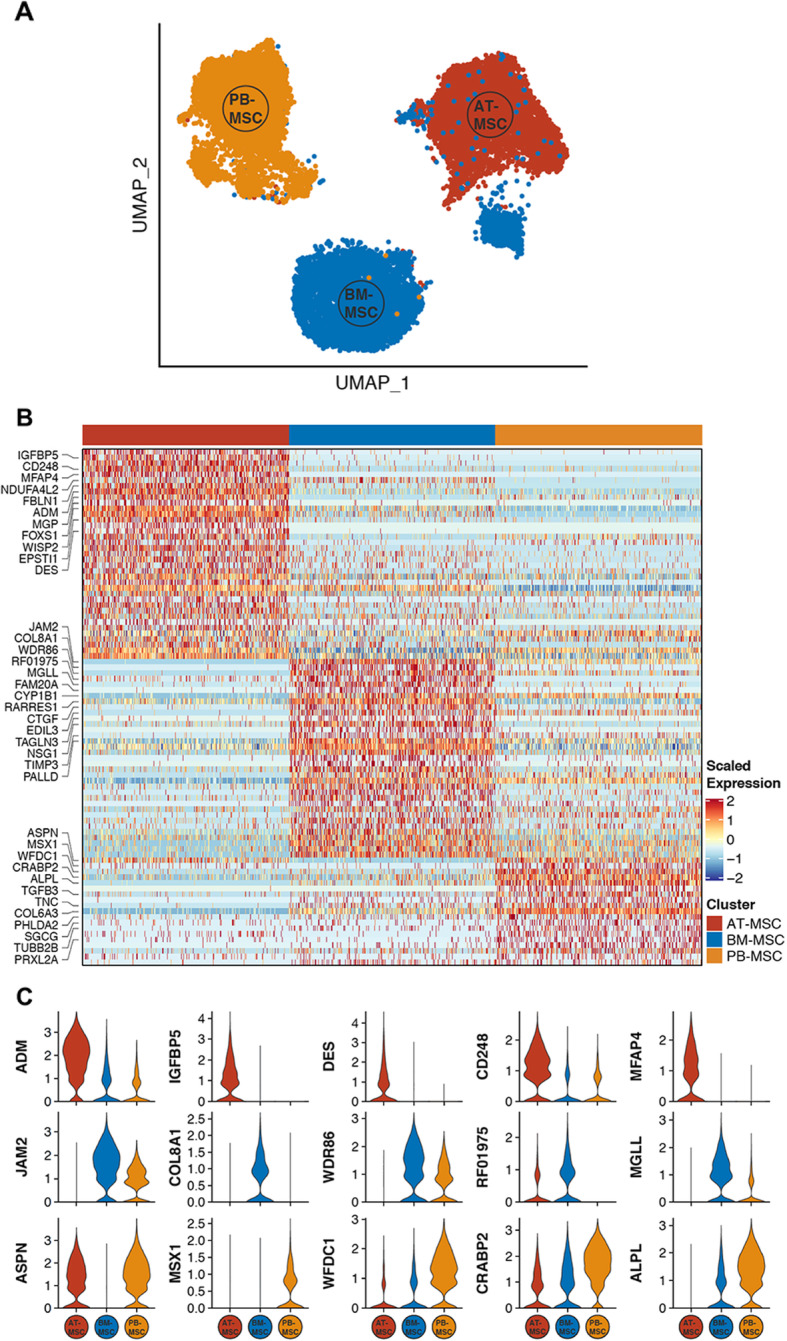
Single-cell RNA sequencing (sc-RNAseq) data reveal inter-source variation of equine mesenchymal stromal cells (MSCs). **a** UMAP plot depicting clustering of MSCs isolated from donor-matched adipose tissue (AT), bone marrow (BM), and peripheral blood (PB). **b** Heatmap showing significant differentially expressed genes (DEGs) (adjusted *p*-value < 0.05, log_2_ fold change > 1 for each tissue source versus all others, percentage of tissue source with detectable expression of gene > 25%) between tissue sources. Top 10 (ranked by log_2_ fold change) genes per tissue source are labeled with gene symbol. For visualization purposes, 250 cells (columns) were randomly selected from each tissue source. **c** Violin plots of the expression levels of the top 5 DEGs (ranked by log_2_ fold change) in MSCs isolated from each tissue source. See also Additional files 1 and 2

To characterize additional defining transcriptional features of MSCs derived from different tissue sources, we analyzed scRNA-seq data from all three tissue sources in combination. Blinded to tissue origin, unsupervised clustering partitioned 3 distinct cell groups. Post-hoc assignment of sample source revealed that cells predominantly cluster by tissue of origin (Fig. 2a). Grouping all single MSCs derived from a given tissue source and modeling for sub-cluster composition, differential gene expression (DGE) analysis between tissue sources revealed distinct gene expression patterns across MSCs isolated from each source (Fig. 2b). We detected 37, 35, and 20 differentially expressed genes (log_2_ fold change greater than 1, adjusted *p* value less than 0.05, and percentage of tissue source with detectable expression of gene > 25%) in AT-, BM-, and PB-derived MSCs, respectively (Additional file 1). Gene ontology (GO) term enrichment analysis indicated that top ranked GO terms associated with AT-MSCs are GO:0043567 insulin-like growth factor regulation and GO:000823 cell proliferation; with BM-MSCs are GO:0030198 extracellular matrix organization and GO:007155 cell adhesion; and with PB-MSCs are GO:0042476 odontogenesis, GO:0048705 skeletal system development and GO:0016114 small molecule synthesis (Additional file 2). Expression levels of the top five differentially expressed genes (ranked by fold change) from each tissue source are presented in Fig. 2c. The expression patterns of 6 genes from this group were validated by RT-PCR (Figure S4). Furthermore, these transcripts were analyzed in matched AT-, BM- and PB-MSC isolated from 2 additional donor horses (Figure S4). Overall, tissue specific expression patterns were maintained from horse to horse, with the exception of monoglyceride lipase *(MGLL)* and cellular retinoic acid binding protein 2 *(CRABP2),* which were differentially expressed in MSC isolated from Horse 2.

### Junctional adhesion molecule 2 (JAM2) modulates the cell motility phenotype of BM-derived MSCs

Based on marked and distinct differential expression by scRNA-seq, and involvement in processes related to cell motility, we decided to focus on junctional adhesion molecule 2 (*JAM2)* and to evaluate its contribution to inter-source MSC differences. *JAM2* encodes the JAM-2/JAM-B protein and was found by scRNA-seq to be expressed at significantly higher levels in BM-derived MSCs relative to both PB-derived MSCs and AT-derived MSCs (Fig. 2c). Using quantitative RT-PCR, we confirmed the expression patterns of *JAM2* in MSCs from these three different tissue sources (Figure S3B). JAM-2 is known to be expressed on endothelial cells and hematopoietic stem cells (HSC), as well as cancer cells, and to play a role in various processes involving proliferation, adhesion, migration, and invasion [49–51].

**Fig. 3:**
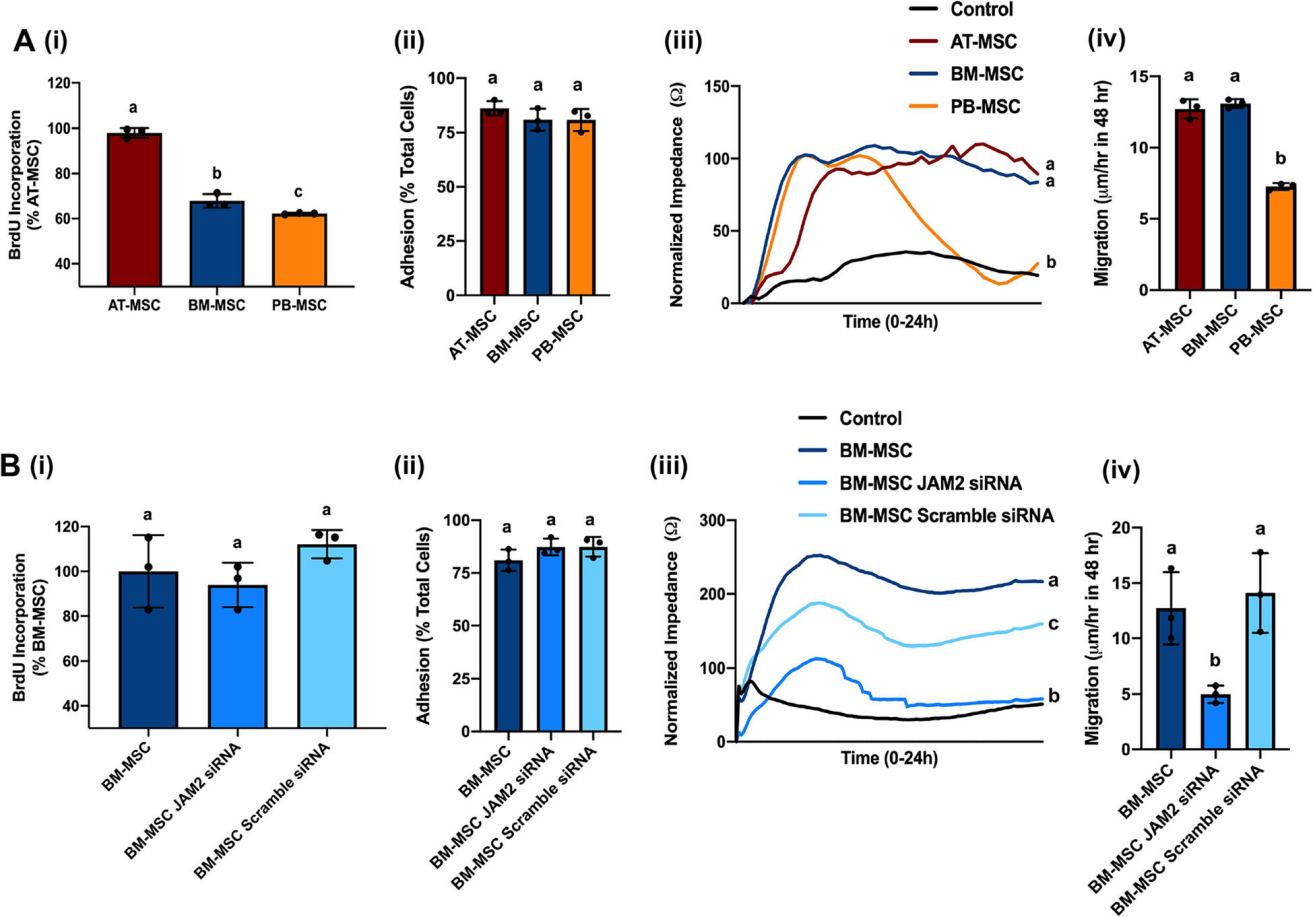
Knock down of junctional adhesion molecule 2 (*JAM2)* in bone marrow (BM)-derived mesenchymal stromal cells (MSCs) alters cell motility. **a** Base line measurements in MSCs derived from donor-matched adipose tissue (AT), bone marrow (BM), and peripheral blood (PB) of cell proliferation rate, as determined by incorporation of 5-Bromo-2′-deoxyuridine (BrdU) incorporation **(i);** cell adhesion as measured by centrifugation assay **(ii)**; cell invasion through an equine endothelial cell (EC) monolayer, as determined by electric cell impendence sensing (ECIS) **(iii)**; and cell migration, as determined by an in vitro scratch assay **(iv)**. **b** Measurements in BM-derived MSCs that were either not transfected or transfected with *JAM2*-specific siRNA or scramble siRNA (control) of cell proliferation rate, as determined by incorporation of 5-Bromo-2′-deoxyuridine (BrdU) incorporation **(i);** cell adhesion as measured by centrifugation assay **(ii)**; cell invasion through an equine endothelial cell (EC) monolayer, as determined by electric cell impendence sensing (ECIS) **(iii)**; and cell migration, as determined by an in vitro scratch assay **(iv)**. Significant differences are depicted by different letters, *n* = 3. Data are presented as the mean ± standard deviation. *P* < 0.05

Interestingly, JAM-2 expressed on BM-derived stromal cells has been shown to interact with JAM-C on HSC, regulating the migration of HSC progenitors in and out the BM in vivo [52], but the ways in which JAM-2 expression levels on cultured MSC modulates proliferation and motility of these cells themselves have not been explored. To begin exploring the role(s) of JAM2 in MSC biology, we first examined baseline levels of JAM-2-associated functions in MSCs from the three donor-matched tissue sources. We found a significant difference in MSC proliferation, as assessed by 5-Bromo-2′-deoxyuridine (BrdU) incorporation, with AT-derived MSCs showing the highest proliferation, followed by BM- and PB-derived MSCs (Fig. 3a(i)), but we did not observe any significant differences in adhesion strength between MSCs from these three sources (Fig. 3a(ii)). Electric cell-substrate impedance sensing (ECIS) assays measuring invasion showed that PB-, but not AT- and BM-, derived MSCs could invade a monolayer of primary equine endothelial cells (ECs), as shown by a significant drop in impedance indicating a disruption of the equine EC monolayer (Fig. 3a(iii)). Finally, we observed that AT- and BM-derived MSCs migrated significantly faster using scratch assays when compared to PB-derived MSCs (Fig. 3a(iv)).

To assess the potential role of JAM-2 in these processes, we used RNA interference (RNAi) to knock down *JAM2* expression in BM-derived MSCs, the source with the highest levels of *JAM2* (Fig. 2c and S3B). JAM2 expression was significantly reduced in BM-derived MSCs transfected with *JAM2*-specific siRNA, but not in BM-derived MSCs transfected with scramble siRNA, when compared to non-transfected control MSCs, both on mRNA and protein level (Figure S3C, D).

Repeating the functional assays with *JAM2*-siRNA-transfected BM-derived MSCs showed that the knock down of *JAM2* did not significantly affect BM-derived MSCs proliferation (Fig. 3b(i)), nor did it change adhesion strength (Fig. 3b(ii)), indicating that JAM-2 plays a redundant role in these functions in BM-derived MSCs. Consequently, the observed inter-source difference in proliferation at baseline (Fig. 3a(i)) is most likely mediated by other genes/proteins. In contrast, knockdown of *JAM2* resulted in increased invasion capacity (Fig. 3b(iii)) and decreased migration (Fig. 3b(iv)) of BM-derived MSCs, demonstrating that JAM-2 modulates the cell motility phenotype of BM-derived MSCs, a characteristic that may be therapeutically beneficial. Moreover, *JAM2* knockdown in BM-derived MSCs rendered these cells functionally more similar to PB-derived MSCs (Fig. 3a), the MSC source with significantly lower expression of *JAM2* as determined by scRNA-Seq (Fig. 2c). Still, *JAM2* expression alone cannot account for these two motility functions, since BM- and AT-derived MSCs showed similar levels of invasion and migration (Fig. 3a(iii)&(iv)) despite significantly different expression levels of *JAM2* between these two sources (Fig. 2c). It will, therefore, be of interest in future studies to explore the differential expression of additional genes with migratory functions that were identified in our scRNA-Seq analysis, and how altering their expression, alone or in combination, influences MSC motility. For example, insulin-like growth factor binding protein 5 (*IGFBP5*), which is highly expressed on AT-derived MSCs and showed low levels of expression on BM- and PB-derived MSCs (Fig. 2c), has been reported to either stimulate or inhibit cell migration, depending on the cell type it is expressed on [53, 54], and thus, could be an interesting candidate for follow up.

Collectively, these results demonstrate that *JAM2* is involved in cell invasion and migration of BM-derived MSCs. In addition to differential gene expression, we suggest that inter-source heterogeneity of MSCs translates into biologically relevant MSC functions, such as those related to cell motility.

### scRNA-seq data reveal intra-source variation of equine MSCs isolated from donor-matched tissue sources

**Fig. 4:**
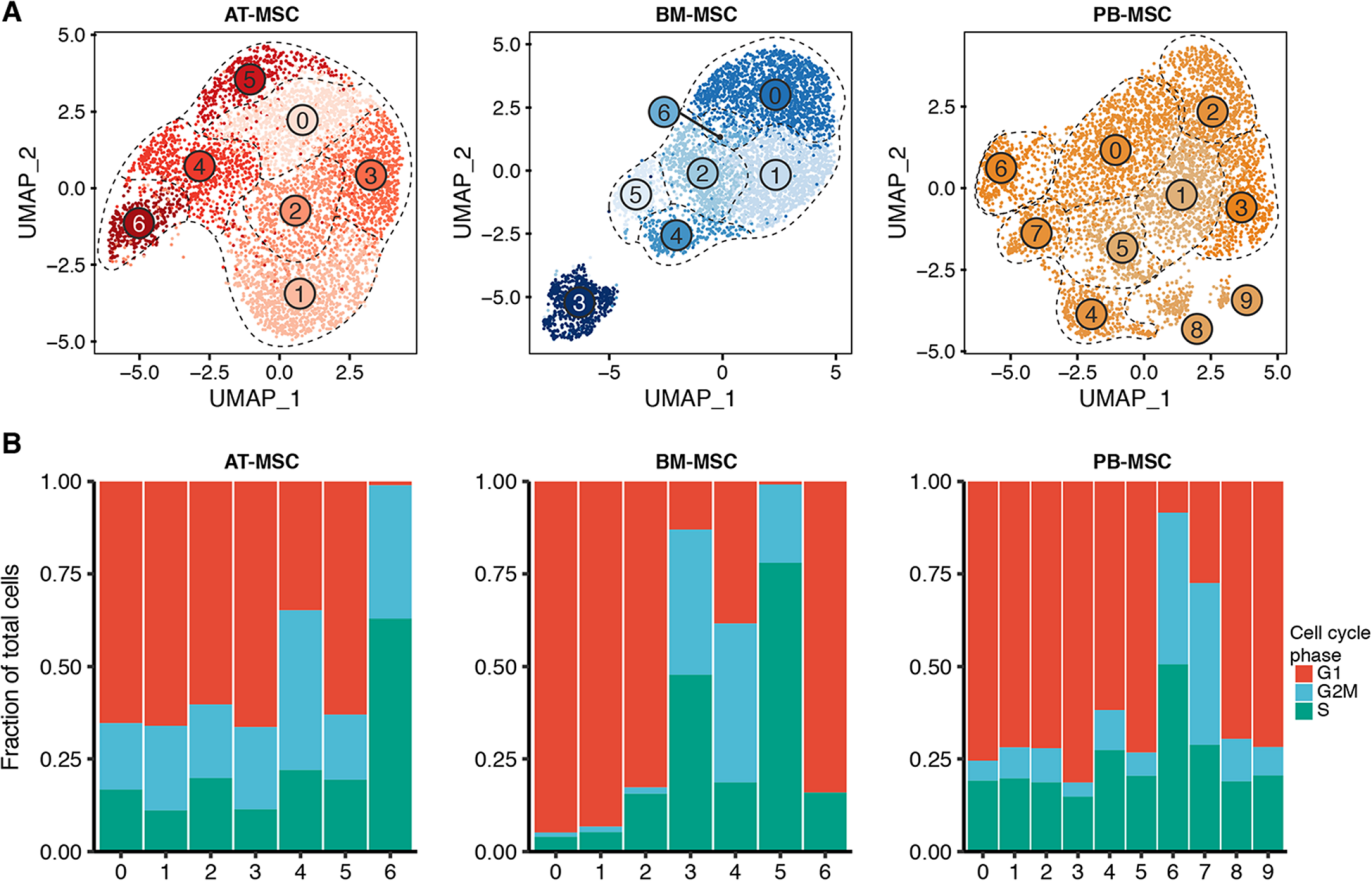
Single-cell RNA sequencing (sc-RNAseq) data reveal intra-source variation of equine mesenchymal stromal cells (MSCs). **a** UMAP plots of MSCs isolated from AT, BM and PB showing intra-source variation. **b** Frequency of cell cycle classification per cluster in MSCs derived from each tissue source; AT, BM and PB. See also Additional files 3, 4, 5, 6, 7, 8

**Fig. 5:**
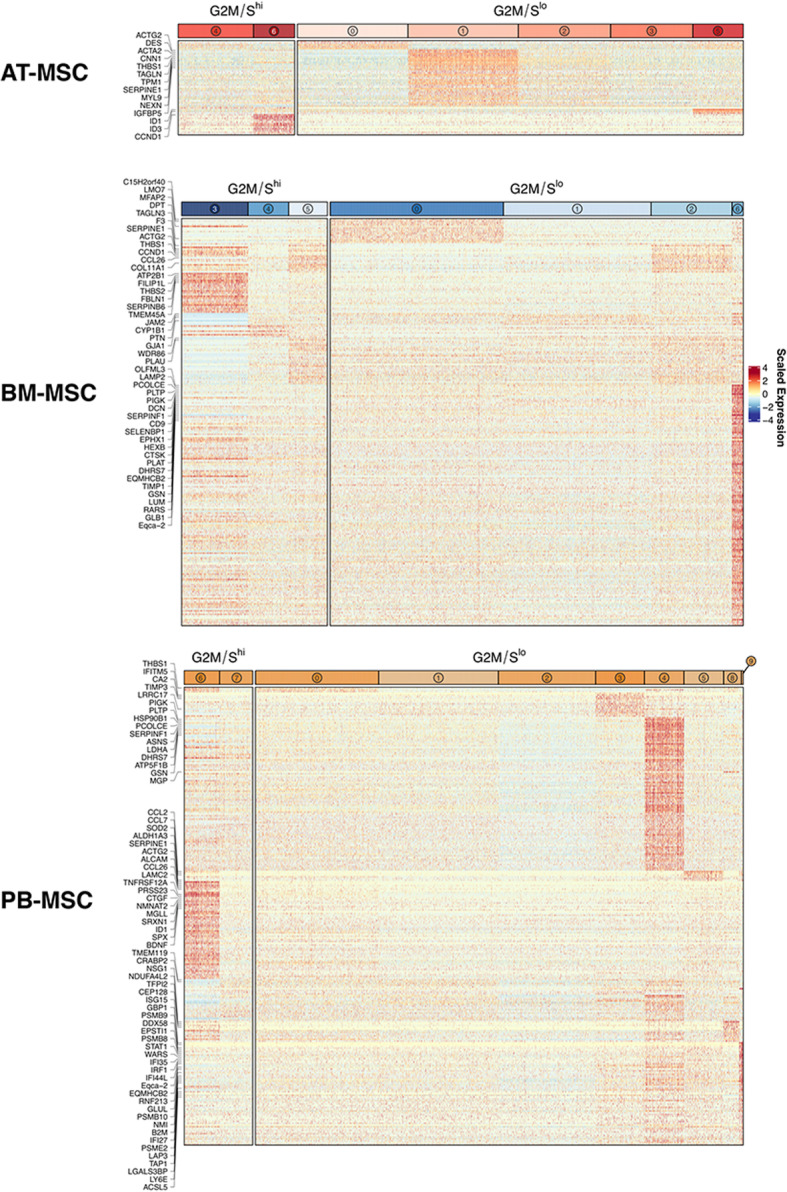
Equine mesenchymal stromal cells (MSCs) isolated from 3 donor-matched tissue sources exhibit intra-source transcriptional heterogeneity. Heatmaps of differentially expressed genes (DEGs) (adjusted p-value < 0.05, log_2_ fold change > 1 for each cluster versus all other clusters within tissue source/cell cycle classification, percentage of cluster with detectable expression of gene > 25) for AT-, BM- and PB-derived MSCs, revealing intra-source transcriptional heterogeneity. Individual heat maps were grouped by cluster cell cycle classification (G2M/S^hi^ and G2M/S^lo^) / corresponding DGE analysis. DEGs with log_2_ fold change > 1.5 are labeled with gene symbol

In our initial analysis, we observed that clustering was considerably influenced by expression of cell cycle-related genes (data not shown), a phenomenon that has been previously reported in scRNA-seq studies profiling cells in culture [55]. We, therefore, accounted for “cell cycle effect” in data normalization (details in *Methods*). In independent analyses (i.e. per tissue source), unsupervised clustering partitioned the AT-derived MSC and the BM-derived MSCs into 7 clusters each, and the PB-derived MSCs into 10 clusters (Fig. 4a). Despite mitigating the effects of cell cycle gene expression, we continued to observe some clusters apparently defined by gene expression patterns consistent with proliferation (Fig. 4b). Therefore, we further analyzed G1 clusters (G2M/S^lo^) separately from G2M/S clusters (G2M/S^hi^) in order to identify differential gene expression patterns across clusters independent of cell cycle classification (Additional files 3, 4, 5, 6, 7, 8). Under this grouping strategy, differential gene expression testing revealed appreciable intra-source transcriptional heterogeneity in MSCs derived from AT, BM and PB, albeit to a lesser extent in the AT-derived MSC (Fig. 5). For 3 of 5 G2M/S^lo^ AT-derived MSC clusters, we detected 5 (cluster 0), 32 (cluster 1) and 3 (cluster 5) DEGs, while we detected no DEGs in clusters 2 and 3 (log_2_ fold change greater than 1, adjusted *p* value less than 0.05, and percentage of tissue source with detectable expression of gene > 25%). Of the G2M/S^hi^ AT-derived MSC clusters, we detected 3 and 13 DEGs for cluster 4 and cluster 6, respectively (Additional file 3). For G2M/S^lo^ BM-derived MSC clusters, we detected 14 (cluster 0), 1 (cluster 1), 16 (cluster 2) and 140 (cluster 6) DEGs. Of the G2M/S^hi^ BM-derived MSC clusters, we detected 28, 15, and 38 DEGs for cluster 3, cluster 4 and cluster 5, respectively (Additional file 4). For 6 of 8 G2M/S^lo^ PB-derived MSC clusters, we detected 3 (cluster 0), 14 (cluster 4), 89 (cluster 4), 6 (cluster 5), 16 (cluster 8) and 67 (cluster 9) DEGs. No differentially expressed genes were detected in cluster 1 or 2 in PB-derived MSCs. Of the G2M/S^hi^ PB-derived MSC clusters, we detected 60 (cluster 6) and 34 (cluster 7) DEGs (Additional file 5).

To examine putative biological functions of detected clusters, we conducted GO term enrichment analysis for each cluster within each tissue source. Of clusters where we detected significant enrichment in intra-source comparisons, top ranked (per cluster) GO terms within BM-derived MSC cultures were GO:0016477 cell migration (cluster 2) and GO:002544 chronic inflammatory response (cluster 3). Within the PB-derived MSC culture, top ranked GO terms were GO:0006457 protein folding (cluster 4), GO:0050920 regulation of chemotaxis (cluster 6), GO:0044283 small molecule biosynthetic process (cluster 7), GO:0001525 angiogenesis (cluster 8) and GO:0034097 response to cytokine (cluster 9). We did not detect any significantly enriched GO terms in AT-derived MSC clusters, suggesting minimal transcriptional differences across detected clusters. Complete GO term enrichment results are reported as Additional files 6, 7, 8.

### Clonal heterogeneity of PB-derived MSCs reveals functional differences in chemoattractant capacity

**Fig. 6:**
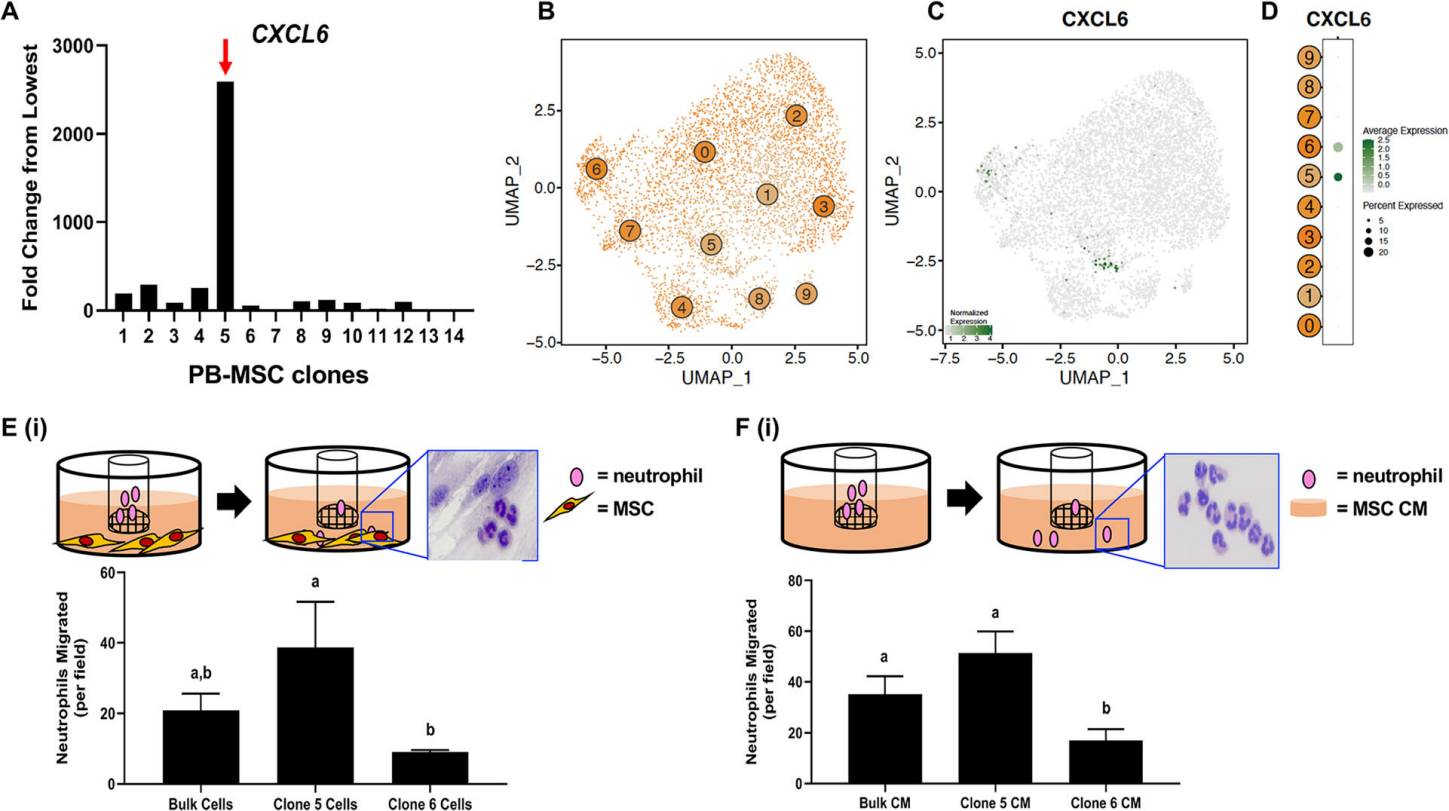
Intra-source heterogeneity of peripheral blood (PB)-derived mesenchymal stromal cells (MSCs) translates into varying chemoattractant capacity. **a** C-X-C motif chemokine ligand 6 *(CXCL6)* expression in 14 clones generated from a bulk PB-derived MSC culture, as determined by qRT-PCR. **b** UMAP representation of PB-derived MSC clusters. Points are colored by unsupervised clustering assignment. **c** *CXCL6* RNA expression pattern in PB-derived MSCs. Expression values are scaled from 2nd to 98th percentile of gene expression across all plotted cells. **d** *CXCL6* RNA expression across PB-derived MSC clusters. Dot size is proportional to number of cells with detectable expression of *CXCL6.* Dot color intensity indicates gene expression values scaled across plotted clusters. **e** Schematic of the neutrophil chemotaxis assays, with insert showing hematoxylin stained neutrophils and MSC, after neutrophil migration **(i)** and quantification of neutrophil migration toward the bulk PB-derived MSC population, clone 5 MSCs, and clone 6 MSCs **(ii)**. **f** Schematic of the neutrophil chemotaxis assays, with insert showing hematoxylin stained neutrophils after migration **(i)** and quantification of neutrophil migration toward the conditioned medium (CM) from the bulk PB-derived MSC population, clone 5 MSCs, and clone 6 MSCs **(ii).** Significant differences are depicted by different letters, *n* = 3. Data are presented as the mean ± standard deviation. P < 0.05

Among the GO terms significantly enriched in PB- and BM-MSC analyses, we noted several terms associated with cell migration and chemotaxis. We, therefore, explored possible functionally intra-source variation in MSC chemotactic activity. To this end, we used an in vitro limiting dilution cell cloning assay to generate clonal PB-derived MSC lines to be used for downstream functional chemotaxis assays [46] (Figure S1). Fourteen clonal cell lines from individual PB-derived MSCs were generated and evaluated by RT-PCR for expression of various chemotaxis-related genes, as informed by the scRNA-seq analysis (data not shown). We found C-X-C motif chemokine ligand 6 (*CXCL6)*, a molecule involved in the recruitment of granulocytes, to be expressed at a significantly higher level in clone 5 relative to the other clones (Fig. 6a). This was consistent with detection *of CXCL6* expression in a minority of cells in the PB-derived MSC population by scRNA-Seq (Fig. 6b-d). As a follow up, we performed an in vitro chemotaxis assay with clone 5 MSCs, comparing the neutrophil attractant activity of CXCL6^hi^ MSCs to CXCL6^low^ MSCs (clone 6, Fig. 6a). Briefly, MSCs were added to the lower wells of a transwell plate with a 3 μm pore insert which was seeded with equine neutrophils (Fig. 6e(i)). We observed that CXCL6^hi^ MSCs stimulated neutrophil migration to significantly higher levels compared to CXCL6^low^ MSCs, while the bulk, original MSC culture stimulated neutrophil migration to intermediate levels (Fig. 6e(ii)). Similar results were observed when these experiments were performed with conditioned medium (CM) collected from CXCL6^hi^ MSCs (Fig. 6f), indicating that the effects of MSCs on chemotaxis do not require direct cell contact.

These results demonstrate that *CXCL6* expression levels in MSCs are correlated with increased neutrophil migration in vitro and provide proof-of-concept that intra-source heterogeneity in PB-derived MSCs translates into biologically relevant functions, such as those related to immune modulation.

## Discussion

To the best of our knowledge, this study is the first to use single-cell RNA sequencing (scRNA-seq) to compare expression profiles of mesenchymal stromal cells (MSCs) isolated from 3 tissue sources from a single donor. Gene expression profiling at single cell resolution indicate that MSCs derived from different tissue sources are transcriptionally distinct, and that MSCs derived from the same tissue source exhibit variation in gene expression as well. Importantly, we demonstrate that this inter- and intra-source transcriptomic heterogeneity corresponds with differences in biological function, with individual cells/populations exhibiting varied phenotypes.

It is generally accepted, albeit not very well understood, that MSCs isolated from different tissue sources vary phenotypically [56–58] and consequently, have distinct therapeutic capacities [59]. This study confirmed inter-source heterogeneity across cultures derived from three tissue sources from the same donor. All samples were processed in parallel at the same time, and although we cannot fully rule out the possibility that the observed inter-source differences could be due to technical batch effects, we believe this to be unlikely based on differential gene expression patterns. Additionally, we showed that junctional adhesion molecule 2 *(JAM2)* expression levels contribute to differences in MSC migration and invasion. The importance of MSC motility when considering these cells for therapy is well appreciated [60, 61]. Ideally, after being injected into a patient, MSCs migrate to the site of damaged tissue, where they contribute to repair by secreting active factors that recruit immune cells and stimulate resident cells [62, 63]. For cancer therapy, optimal MSCs for treatment are considered those with invasive properties so that they can integrate into tumors [64]. Using knock-down experiments of *JAM2* as proof-of-concept, we determined that this molecule contributes to migration and invasion of BM-derived MSCs. Although the role of JAM-2 has been well-explored in the context of its interaction with JAM-C on hematopoietic stem cells (HSC), resulting in the regulation of the migration of HSC progenitors in and out the BM in vivo [52], its role on migration and other JAM-2-associated functions of MSCs had not been explored until now.

In our initial analyses, we found that a considerable degree of intra-source transcriptional heterogeneity could be attributed to genes associated with cell cycle. This influence of genes involved in cell cycling was noted previously by another group using scRNA-seq to study human MSCs [65] but was not directly addressed in their analysis. Upon accounting for cell cycle phase (as assigned by gene expression patterns), we observed intra-source heterogeneity of BM- and PB-derived MSCs. Differential gene expression testing between clusters revealed low numbers of differentially expressed genes detected in AT-derived equine MSCs, a result that is similar to one obtained in a study using human AT-derived MSCs [20]. In that study, AT-derived MSCs were found to exhibit less transcriptomic heterogeneity when compared to donor-matched BM-derived MSCs. Our current work now expands on this finding by including PB-derived MSCs. These data add to the growing list of similarities between MSCs from human and equine origin [66].

In addition to intra-source transcriptional heterogeneity detected in our scRNA-seq analyses, we also observed intra-source differences in MSC functions, i.e. chemoattractive activity. MSC-based immunomodulation is considered highly relevant therapeutically and MSCs are primarily studied in human medicine for treatment of immune-related diseases and as anti-cancer therapies [67–69]. We found that clonal cell lines derived from individual PB-derived MSCs exhibited varying expression levels of C-X-C motif chemokine ligand 6 (*CXCL6)* and that this variation in expression directly correlated with neutrophil attractant activity. In this regard, it is worth noting that human progenitor cell derived CXCL6 has been explored as a therapy [70] and that equine and human MSCs can be primed to specifically increase CXCL6 production with the goal of improving therapeutic outcomes [71, 72]. To obtain these clonal cell lines, we separated cells using a technique employed by other research groups working with MSCs [73, 74]. Thus, it can be envisioned that clinical efficacy of MSC cultures may be enhanced by screening for clonal cell lines that express a specific immunomodulatory expression pattern beneficial for a specific disease. Moreover, if such a clonal cell line of interest would express a distinct combination of genes encoding cell surface markers, then MSCs with that particular profile could be captured by flow cytometric cell sorting from a bulk MSC mixed culture. This approach has been used previously to enrich human BM-derived MSCs for CD146^+^ cells, with the goal of establishing a culture with enhanced osteogenic differentiation potential [75]. It has also been shown effective for the isolation of CD45^−^/Ter119^−^/Sca-1^+^ cells from primary mouse BM-derived MSCs. These markers are indicative of mesenchymal progenitors, allowing them to be separated from contaminating hematopoietic cells [76]. Indeed, our scRNA-seq results expand the list of potential surface markers to isolate MSCs from different tissue sources.

While our group and others have begun to explore MSC heterogeneity, the sources and degree of this heterogeneity are not well understood and widely debated [77, 78]. It has been proposed that MSC populations may either be comprised of a mixture of cell types with different cell fates or that they are cells of one particular type that exhibits subtler cell-to-cell variation. In addition, heterogeneity can also be introduced by cell environment, where MSCs derived from different tissue sources may be similar in origin but primed by the tissue they reside in to exhibit distinct functions. Further experimental studies are necessary to fully understand variation in MSCs and how that variation impacts MSC function.

The horse is not only an ideal large animal for the collection of donor matched MSCs from different tissues for transcriptomic and functional in vitro studies, but it is also a physiologically highly relevant species that can be used for studies to test MSC activities in vivo. For example, a well-established equine skin wound model was used for treatment with endothelial colony forming cells and local immune cell responses were assessed, in part, based on neutrophil density at the wound site [79]. We propose a similar model could be used to evaluate the efficacy of MSC clones exhibiting differing levels of *CXCL6* expression on wound healing, and to specifically determine if MSC-secreted CXCL6 affects neutrophil migration to damaged tissue by assessing the level of neutrophil migration. Another equine model, in which labeled MSCs were injected into surgically created superficial digital flexor tendon (SDFT) lesions of horses and tracked for up to 9 weeks post-treatment with low-field magnetic resonance imaging [80] could, in theory, be used to evaluate the in vivo importance of JAM-2 for MSC motility by injecting labeled MSCs with stably knocked-down or over-expressed *JAM2*. It would be of interest to design such future studies, based on the relevance of equine skin wound and SDFT lesion models for human wound management and orthopedic injuries [26, 27] respectively.

## Conclusions

Taken together, our work demonstrates that single-cell transcriptomic data can provide the rationale for functional studies that will allow for (i) a better understanding of the cellular heterogeneity that imparts specific therapeutically beneficial properties on MSC and (ii) the development of methods to capture and expand specific MSCs that exhibit these properties. These advances will accelerate MSC therapy to move from bench to bedside.

## Supplementary Information

**Additional file 1.**

**Additional file 2.**

**Additional file 3.**

**Additional file 4.**

**Additional file 5.**

**Additional file 6.**

**Additional file 7.**

**Additional file 8.**

**Additional file 9: Figure S1.** Cloning procedure used to generate mesenchymal stromal cell (MSC) lines from single cells. MSC isolated from peripheral blood (PB) were labeled with CellTracker to visualize single individual cells. Cells were plated at a frequency of 0.5 cells per well in 96-well plate wells, and wells containing single cells were identified. Upon confluency, those clones that started with single cells were expanded and collected for further analysis.

**Additional file 10: Figure S2.** Flow cytometry plots of mesenchymal stromal cell (MSC) protein expression patterns. **a** Plots of proteins expressed by MSC. **b** Plots of proteins not expressed/expressed at low levels by MSC. Colored histograms indicate fluorescence of MSC labeled with protein-specific antibodies. Gray histograms indicate fluorescence of MSC labeled with appropriate immunoglobulin isotypes as controls.

**Additional file 11: Figure S3.** **a** UMAP plot of CD34 transcripts detected in MSCs by scRNA-seq. **b** *JAM2* expression in MSCs isolated from donor-matched adipose tissue (AT), bone marrow (BM), and peripheral blood (PB) by RT-PCR. JAM2 expression of BM-derived MSCs that were either not transfected or transfected with *JAM2*-specific siRNA or scramble siRNA (control) by RT-PCR **c** and Western blot **d** Representative images of blots are included as well **(ii).** Significant differences are depicted by different letters, *n* = 3 and *n* = 2 for RT-PCR and Western blots, respectively. Data are presented as the mean ± standard deviation. *P* < 0.05.

**Additional file 12: Figure S4.** Validation of mesenchymal stromal cell (MSC) gene expression patterns detected using Single-cell RNA sequencing (sc-RNAseq). Graphs of gene expression patterns in adipose tissue (AT), bone marrow (BM), and peripheral blood (PB) MSC isolated from 3 donor horses as determined by RT-PCR. MSC isolated from Horse 1 were used for sc-RNAseq.

## Data Availability

The scRNA-seq dataset supporting the conclusions of this article is available for download from the NCBI Gene Expression Omnibus (http://www.ncbi.nlm.nih.gov/geo/) under accession number # GSE156467.

## Abbreviations

AT: adipose tissue
BM: bone marrow
CRABP2: cellular retinoic acid binding protein 2
CXCL6: C-X-C motif chemokine ligand 6
JAM2: junctional adhesion molecule 2
MGLL: monoglyceride lipase
MSC: mesenchymal stromal cell
PB: peripheral blood
scRNA-seq: single-cell RNA sequencing
