# Supplementary figures and images for: Single-cell RNA sequencing of equine mesenchymal stromal cells from primary donor-matched tissue sources reveals functional heterogeneity in immune modulation and cell motility
Source: Stem Cell Res Ther. 2020 Dec 4;11:524. doi: 10.1186/s13287-020-02043-5 (PMC7716481; doi:10.1186/s13287-020-02043-5)

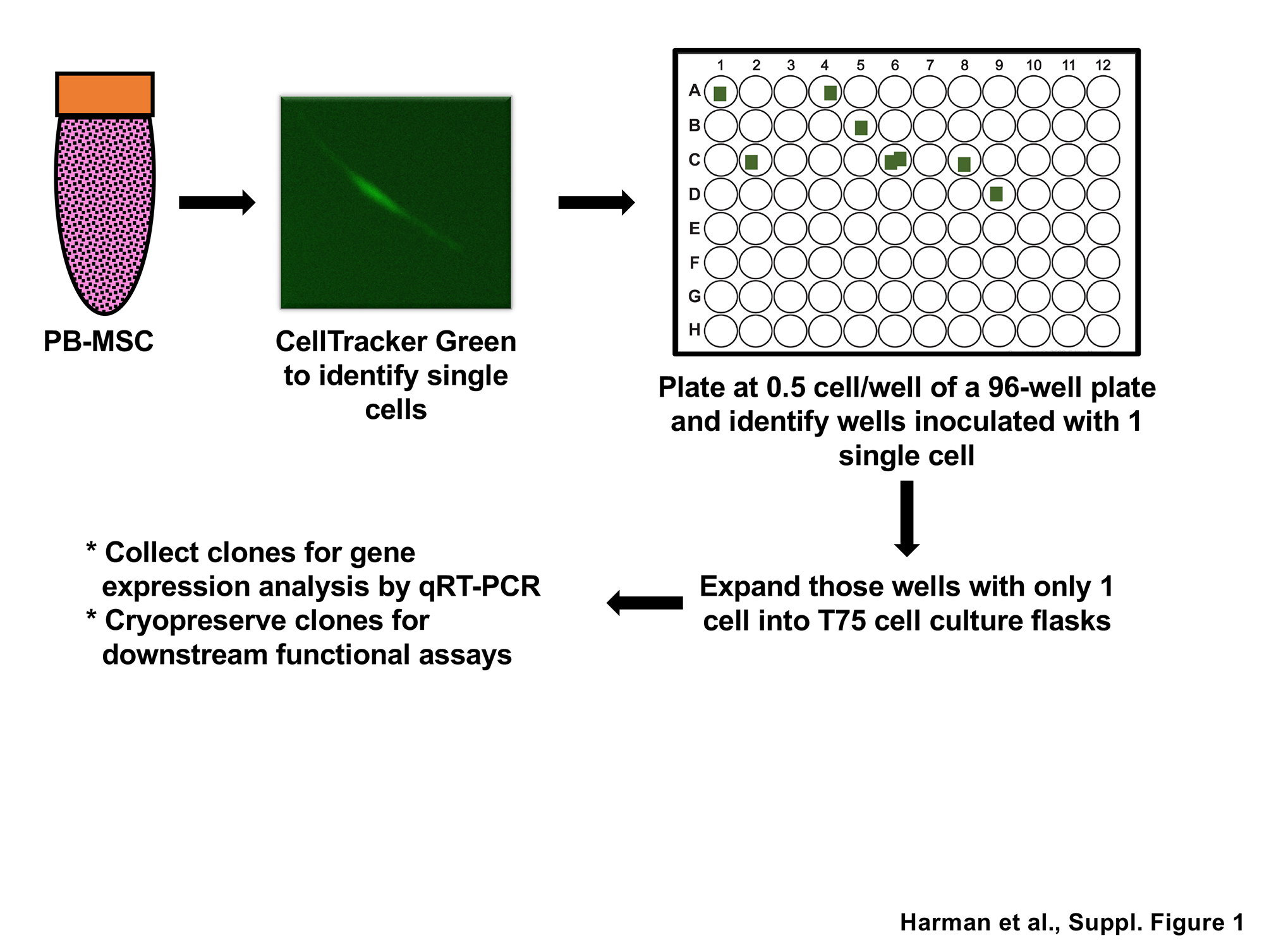

Supplement: Supplementary file 9 — Additional file 9: Figure S1. Cloning procedure used to generate mesenchymal stromal cell (MSC) lines from single cells. MSC isolated from peripheral blood (PB) were labeled with CellTracker to visualize single individual cells. Cells were plated at a frequency of 0.5 cells per well in 96-well plate wells, and wells containing single cells were identified. Upon confluency, those clones that started with single cells were expanded and collected for further analysis. [file 13287_2020_2043_MOESM9_ESM.tif]

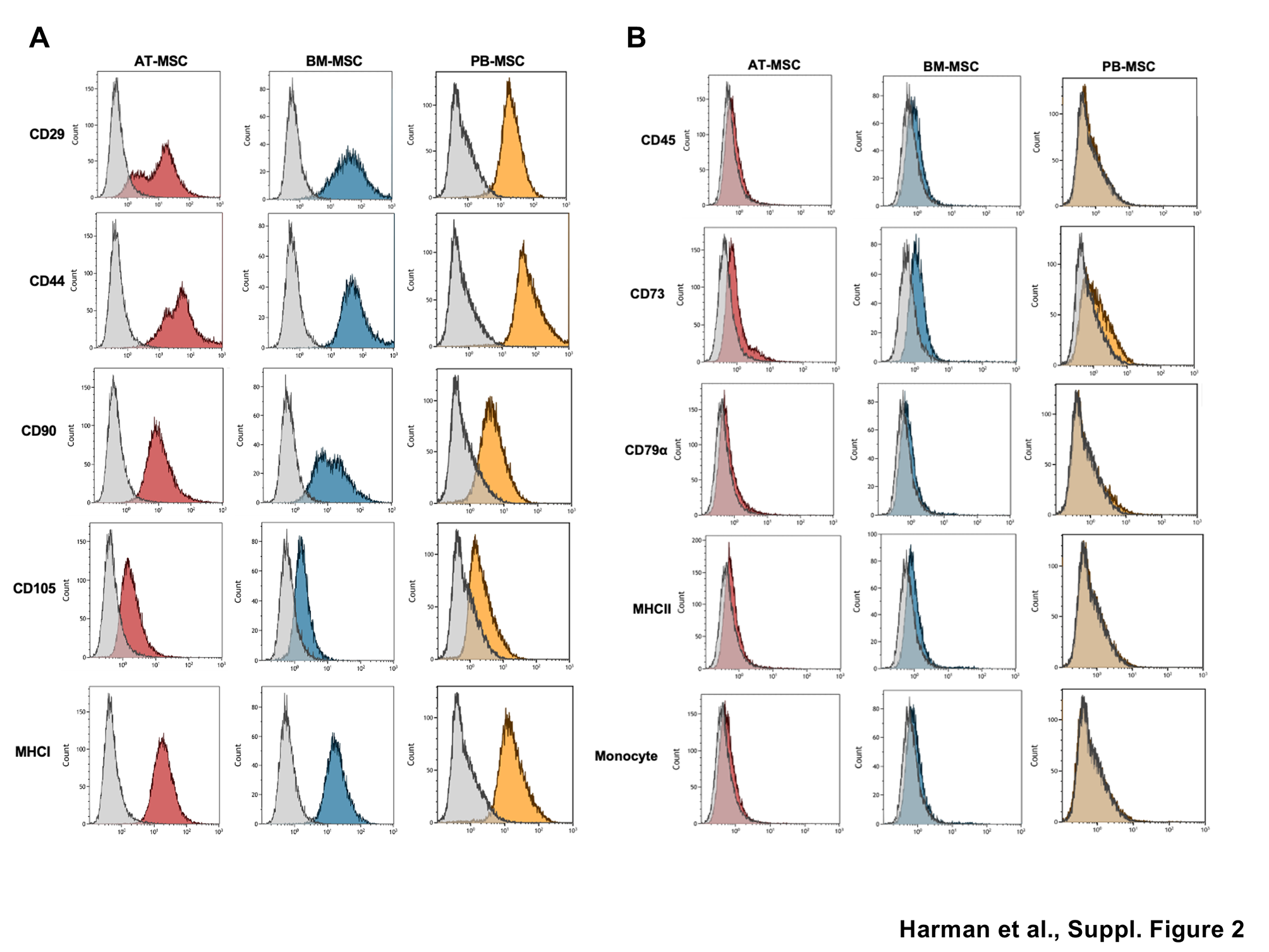

Supplement: Supplementary file 10 — Additional file 10: Figure S2. Flow cytometry plots of mesenchymal stromal cell (MSC) protein expression patterns. a Plots of proteins expressed by MSC. b Plots of proteins not expressed/expressed at low levels by MSC. Colored histograms indicate fluorescence of MSC labeled with protein-specific antibodies. Gray histograms indicate fluorescence of MSC labeled with appropriate immunoglobulin isotypes as controls. [file 13287_2020_2043_MOESM10_ESM.tif]

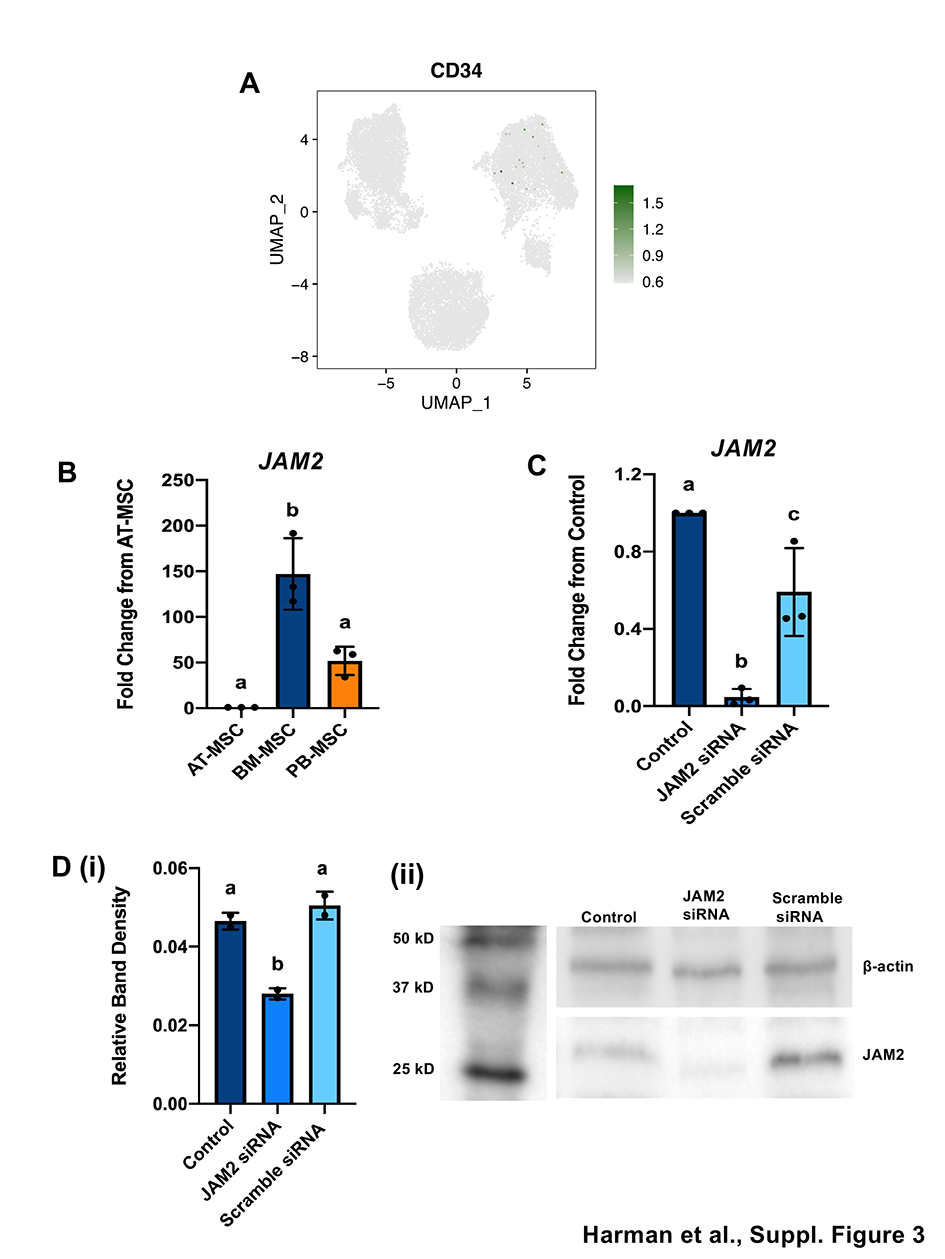

Supplement: Supplementary file 11 — Additional file 11: Figure S3. a UMAP plot of CD34 transcripts detected in MSCs by scRNA-seq. b JAM2 expression in MSCs isolated from donor-matched adipose tissue (AT), bone marrow (BM), and peripheral blood (PB) by RT-PCR. JAM2 expression of BM-derived MSCs that were either not transfected or transfected with JAM2-specific siRNA or scramble siRNA (control) by RT-PCR c and Western blot d Representative images of blots are included as well (ii). Significant differences are depicted by different letters, n = 3 and n = 2 for RT-PCR and Western blots, respectively. Data are presented as the mean ± standard deviation. P < 0.05. [file 13287_2020_2043_MOESM11_ESM.tif]

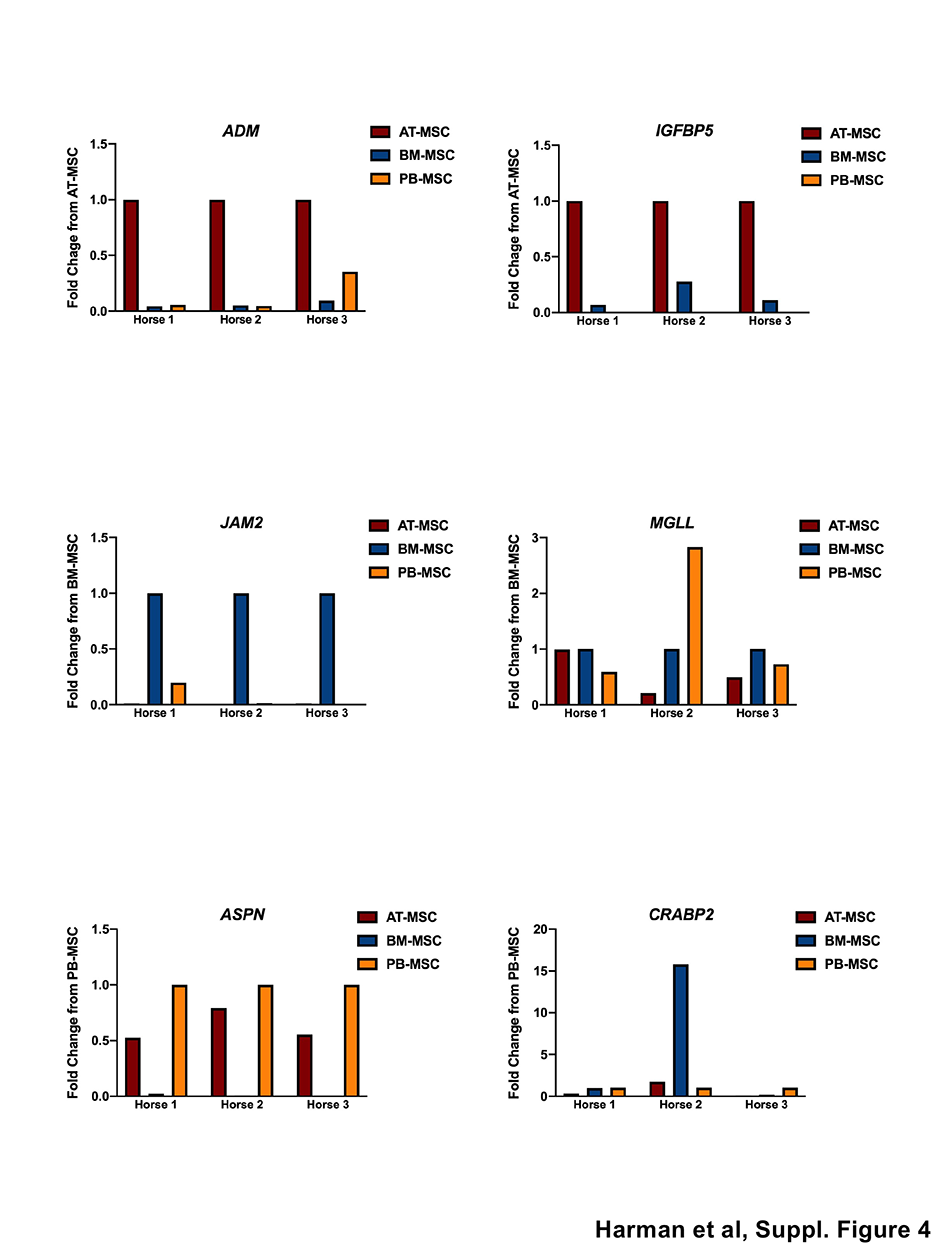

Supplement: Supplementary file 12 — Additional file 12: Figure S4. Validation of mesenchymal stromal cell (MSC) gene expression patterns detected using Single-cell RNA sequencing (sc-RNAseq). Graphs of gene expression patterns in adipose tissue (AT), bone marrow (BM), and peripheral blood (PB) MSC isolated from 3 donor horses as determined by RT-PCR. MSC isolated from Horse 1 were used for sc-RNAseq. [file 13287_2020_2043_MOESM12_ESM.tif]
